# Warm-up durations in a hot-dry climate affect thermoregulation, mean power-output and fatigue, but not peak power in specific soccer repeated-sprint ability

**DOI:** 10.1186/s13102-020-00221-9

**Published:** 2020-12-09

**Authors:** Mohamed Frikha, Nesrine Chaâri, Noureddine Ben Said, Mohammed Shaab Alibrahim

**Affiliations:** 1 Department of Physical Education, College of Education, King Faisal University, Al-Ahsa, Saudi Arabia; 2 Research Laboratory: Education, Motricité, Sport & Santé, UR15JS01, High Institute of Sport and Physical Education, Sfax University, Sfax, Tunisia; 3 Department of Biomechanics and Motor Behavior, College of Sport Sciences and Physical Activity, King Saud University, Riyadh, Saudi Arabia

**Keywords:** Active warm-up, Heat stress, High-intensity effort, Performance, Speed decrement, Football

## Abstract

**Background:**

This study addressed the lack of data on the effect of warm-up (WU) duration in hot-dry climate (~ 30 °C; ~ 18% RH), on thermoregulation, muscular power-output, and fatigue after specific soccer repeated-sprint test (RSA).

**Methods:**

Eleven amateur soccer players participated in a cross-over randomized study and they underwent the Bangsbo repeated-sprint test, after three WU durations (i.e. WU10, WU15 and WU20 min) at 70% of MAV, and on different days. Peak power (PP), mean power (MP) and the fatigue index (FI) were recorded and analyzed. Likewise, heart rate (HR), tympanic temperature (T_tym_), mean body temperature (MBT) and rating of perceived exertion (RPE) were recorded during each session.

**Results:**

The repeated measure ANOVA showed that MP improved after WU15 in comparison to WU10 and WU20 (*p* = 0.04 and *p* = 0.001; respectively). Nonetheless, no significant effect on PP was recorded after all WU durations. FI during RSA increased after WU20 in comparison to WU15 and WU10 (*p* < 0.001 and *p* = 0.003; respectively). Higher RPE values (*p* < 0.001) were recorded after WU15 and WU20 in comparison to WU10 duration. The two-way ANOVA showed higher ΔT_tym_ and ΔMBT values after WU15 and WU20 compared to WU10 (*p* = 0.039 and *p* < 0.001for T_tym_; *p* = 0.005 and *p* < 0.001 for MBT, respectively).

**Conclusions:**

The WU15 at 70% of MAV better assists mean power-output during soccer RSA in hot-dry (~ 30 °C; 18% RH) climate, but not peak power. Reducing WU duration up to 10 min seems to be insufficient to induce beneficial physiological changes necessary for optimizing repeated-sprint performance, while its extension up to 20 min remains detrimental for muscular power and induces higher fatigue.

## Background

Competing in hot climates imposes additional heat stress on participants, increases thermoregulatory strain, reduces performance and accelerates the development of fatigue [1]. Nonetheless, various competition events are held under environmental conditions, ranging from warm to hot climate conditions (25-45 °C) [2], such as the last World Athletics Championship in Doha 2019, the forthcoming Olympic and Paralympic Games in Tokyo 2021, and soccer World Cup in Qatar 2022. Heat stress increases thermal and cardiovascular strain [3], which can affect the choice of the pre-conditioning procedures (i.e. warm-up; WU). Earlier, it was demonstrated that exercising in the heat leads to a greater reliance on muscle glycogen, anaerobic metabolism and post-exercise accumulation of ammonia and blood lactate [4]. Moreover, it was demonstrated that heat exposure leads to an increase in core temperature, which can result in a decrement in high-intensity running performances in women [5] and a reduction in power output during the repeated-sprint in male subjects [3, 6].

Sports performances under heat stress caught the attention of researchers and sports practitioners in the last two decades [1, 3, 6]. However, there is a lack of data related to the effects of warm-up in hot climate on sprint performance [2] and, especially, on soccer RSA. This ability, characterized by the production of maximal short sprint bouts, with brief recovery in between not exceeding 60s [7], corresponds to an essential physical component in soccer competitions [8].

The scientific literature reported no effect of active WU, conducted in either thermoneutral or hot-humid conditions, on intermittent sprint performance [6, 9]. Moreover, soccer players usually performed dynamic WU prior training or competition sessions [10]. This routine was shown to have superior positive acute effects on sprint performance compared to both the FIFA 11+ and Harmoknee programs [10], designed essentially to prevent and reduce soccer-related injuries [11–13].

It was well established that WU procedures aim to bring the body into a ready state before the competition, via temperature-related and non-temperature-related mechanisms [1, 7]. The temperature-related mechanisms consist of a decreased resistance of muscles and joints, a greater release of oxygen from hemoglobin and myoglobin, a speeding of metabolic reactions, increased nerve conduction rate and an increased thermoregulatory strain. The non-temperature-related mechanisms involve an increased blood flow to muscles, an elevation of baseline oxygen consumption, post-activation potentiation, psychological effects and increased preparedness [7]. During the last decade, a large number of researches dealing with WU procedures were conducted [14–16]. However, these findings are still inconclusive and did not give firm conclusions, whether this pre-conditioning training part is practiced in different time-of-day [16], with or without rest interval following the WU [16, 17], among different fitness levels participants [18], or practiced in Ramadan fasting periods [19].

To the authors’ best knowledge, active WU and heat stress effects on specific soccer RSA requires further investigations [2], notwithstanding the existing recommendations supporting the idea of reducing the WU duration in hot climate, when an intermediate and/or intermittent efforts are undertaken [1]. Therefore, the present study aims to examine the effect of different active WU at 70% of MAV, conducted in hot (~ 30 °C) and dry (~ 18% RH) climate, on thermoregulatory responses, muscular power-output, and fatigue during specific soccer repeated-sprint effort. We hypothesize that varying WU duration may arouse an optimal ergogenic effect, necessary to induce better improvement in high-intensity repeated-sprint ability in soccer players.

## Methods

### Participants

Amateur soccer players (*n* = 11; age: 20.5 ± 2.8 yrs.; height: 172.9 ± 4.6 cm; weight: 71.1 ± 5.1 kg; BMI = 23.7 ± 1.1 kg.m^− 2^; MAP = 16.8 ± 0.4 km.h^− 1^; *V*O_2max_ = 55.4 ± 1.1 ml.kg^− 1^.min^− 1^) volunteered to participate to the study. Written informed consent was obtained from all participants after receiving a thorough explanation of the protocol, the benefits and risks involved. The participants were active soccer players with a minimum of 5 years of training experience. They were free from injury and were affiliated in senior (second division) A’Sharqiyah region soccer championship (Eastern Province in Saudi Arabia). They trained systematically 4-5 times a week for an average of 2 h daily. During the experimental period, they were not specially trained for either endurance or sprinting. The study protocol complied with the ethical standards of the 1975 Helsinki Declaration and approved by the King Faisal University Ethic Committee (KFU-180019) before the commencement of the assessments.

### Experimental procedures

Once included, participants were invited to perform the Yo-Yo (Level_1) intermittent recovery test [20]. This test aimed to estimate their maximal “aerobic” velocity (MAV). During the week preceding the experiment, participants familiarized themselves with the Bangsbo repeated-sprint test [8] and the testing procedures in two separate sessions. These familiarizations ensured that participants were fully knowledgeable of the experimental conditions and measurements required. Each familiarization session began with a self-selected WU of 10 min duration followed by the RSA. The best sprint realized (PT) and the total time (TT) were retained, and participants were requested to achieve at least 95% of the time of the first sprint during the testing sessions, otherwise, they will be excluded. Such instruction was imposed to avoid possible pacing during the test [8]. Data from familiarization sessions was retained for rest-retest inter-session reliability, which was set at ICC = 0.882 for TT and ICC = 0.853 for PT. Previously, the coefficients of reliability of the Bangsbo repeated-sprint test were set at 0.91and 0.86 for TT and PT, respectively [21].

The assessments were performed on three separate sessions, in a randomized, counter-balanced order, and over 2 weeks. The assessments were conducted by the same experimenter, outdoor, on flat artificial turf, at the same habitual training time (ie. 4:00–6:00 h PM) and with at least 36 h of recovery in between. Participants were instructed: (i) not to ingest a meal at least 3 h before testing [22] and (ii) not to drink coffee or beverages containing caffeine for at least 8 h before each testing session [23], to avoid any possible effect on muscular power or perceived exertion. However, to avoid the effects of postprandial thermogenesis, 200 to 250 ml of water was allowed.

**Fig. 1:**
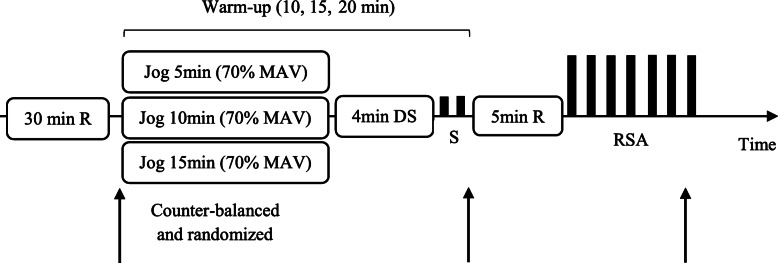
Schematic representation of the experimental design. Arrow: HR, RPE and T; MAV: maximal aerobic velocity; RSA: repeated sprint ability test; R: rest; DS: dynamic stretching; S: 2 × 15 m sprint

Each test session began with 30 min rest in the seated position. After this period, resting heart rate (HR), was recorded using a heart rate monitor (Polar Electro Oy, S410, Hungary). Tympanic temperature (T_tym_) was recorded using a digital thermometer (Braun Thermoscan® IRT 6520 Germany, precision 0.1 °C), while skin (MST) and body (MBT) temperatures were recorded using a digital thermometer (Exacto®, Strasbourg-France, precision 0.1 °C). Following the rest period, the participants completed one of the proposed WU (WU10, WU15 or WU20; Fig. 1).

To avoid a drop in body temperature, an interval of 5 min, between the end of the WU and the onset of the RSA was allowed [16, 17]. The testing sessions took place under similar outdoor conditions. The external temperature (30.6 ± 1.3 °C) and relative humidity (18.7 ± 4.8%) were monitored using a wireless temperature and humidity sensor (Thermo-hygro Oregon Scientific THGR122NX). The participants were recommended to avoid intense activities for 24 h before each test-session, to sleep normally (at least 7 h), and to wear the same sportswear and shoes.

### Rating of perceived exertion (RPE)

The rating of perceived exertion (RPE) was determined using the Borg scale [24], at rest, at the end of the WU (post-WU) and at the end of the RSA (post-RSA). The scale presents a 15-point scale ranging from 6 to 20. The higher the score, the higher the RPE estimation.

### Tympanic (T_tym_), skin (MST) and body (MBT) temperatures

T_tym_ was recorded via tympanic measurements. MST was recorded at four sites of the body: Chest (C), Arm (D), Thigh (H) and Leg (J), as described in Galbraith & Willmott [25]. All temperature measurements were taken three times, with the mean values reported. Mean skin temperature (MST) and mean body temperature (MBT) were calculated using the Burton formulas [26]:

$$ \mathrm{MBT}\ \left({}^{\circ}\mathrm{C}\right)=0.64\ \left({\mathrm{T}}_{\mathrm{tym}}\right)+0.36\ \left(\mathrm{MST}\right);\mathrm{where}\ \mathrm{MST}\ \left({}^{\circ}\mathrm{C}\right)=0.3\ \left(\mathrm{C}+\mathrm{D}\right)+0.2\ \left(\mathrm{H}+\mathrm{J}\right). $$

### Repeated-Sprint ability test (RSA)

A soccer-specific RSA test, known as the Bangsbo repeated-sprint test was used. This test, concluding seven successive maximal sprints with slalom (distance D = 34.2 m), assess the ability to repeat high-intensity, short-duration efforts, with short recovery periods in between as was described in Duarte et al. [8]. Two pairs of photocells (Wireless Training Timer Witty, Microgate, precision 0.001 s, Bolzano, Italy) were positioned on the starting and the finish lines, 0.8 m above the floor. By measuring the time of each sprint, the power output (in Watts) was calculated according to the following eq. [27]:

$$ \mathrm{Power}\ \left(\mathrm{W}\right)=\left(\mathrm{body}\ \mathrm{mass}\times {\mathrm{Distance}}^2\right)/{\mathrm{time}}^3. $$

The peak power (PP), defined as the highest power-output recorded during the 7 sprints, and mean power (MP), defined as the power average of the 7 sprints, were calculated. Likewise, the fatigue index (FI), corresponding to the power decrement was calculated according to the formula [28]:

$$ \mathrm{FI}\ \left(\%\right)=\left[\left(\mathrm{TT}/\mathrm{PT}\times \mathrm{number}\ \mathrm{of}\ \mathrm{sprint}-1\right)\right]\times 100;\mathrm{where}\ \mathrm{TT}\ \mathrm{is}\ \mathrm{the}\ \mathrm{total}\ \mathrm{time}\ \mathrm{and}\ \mathrm{PT}\ \mathrm{is}\ \mathrm{the}\ \mathrm{peak}\ \mathrm{recorded}\ \mathrm{time}. $$

### Warm-up procedures (WU)

Structured according the Jeffreys RAMP model [29], each warm-up session began with a RAISE stage consisting on 5, 10 or 15 min of running at 70% of MAV, followed by 4 min dynamic stretching (DS; ACTIVATE and MOBILIZE) and 2 × 15 m maximal sprint repetitions (POTENTIATE). So that the global WU durations were 10, 15 and 20 min. Participants were instructed to perform the DS stretches. The DS exercises, adopted from previous research [14, 30], involved active and slow movements, without bouncing of antagonist muscles and performed on alternate legs for 30 s, at a rate of approximately 1 stretch cycle every 2 s. The DS consisted of stretches that solicit the major muscle groups involved in maximal sprint: the gastrocnemius, hamstrings, quadriceps, hip flexors and the adductors. All the DS exercises were performed while walking over a distance of 15 m and carried out ~ 10-12 time for each exercise. At the end of the WU, and for neural activation [1], participants were invited to sprint 2 × 15 m, with 25 s recovery in between [27].

### Statistical analyses

All statistical tests were processed using STATISTICA software (StatSoft, France). The normality of data distribution was confirmed by the Shapiro-Wilk W-test. T_tym_, MBT, RPE and HR data’s were analyzed using two-way ANOVA with repeated measures (3 WU duration × 3 measure), while mean power (W) during each sprints test was analyzed using two-way ANOVA with repeated measures (3 WU duration × 7 sprint number). The PP, MP (W.kg^− 1^), and FI (%) were analyzed using a one-way ANOVA (duration WU). When appropriate, significant differences between means were assessed using the Fisher LSD post-hoc test. Furthermore, the effect size “partial η^2^” for significant main effects was calculated. Effect sizes were classified as small (0. 1-0.3), medium (0. 3-0.5), and large (> 0.5) [31]. Statistical significance was set at *p* < 0.05. The power of statistical tests was verified with the G*Power software version (3.1.9.2). Considering the sample size, the significant level of 5% and the partial effect size η^2^, the calculated power analyses (1-β) values were between 0.8 and 1.00 for all variables excepting the PP (1-β = 0.569).

## Results

### Power-output during the running sprint ability test (RSA)

**Fig. 2:**
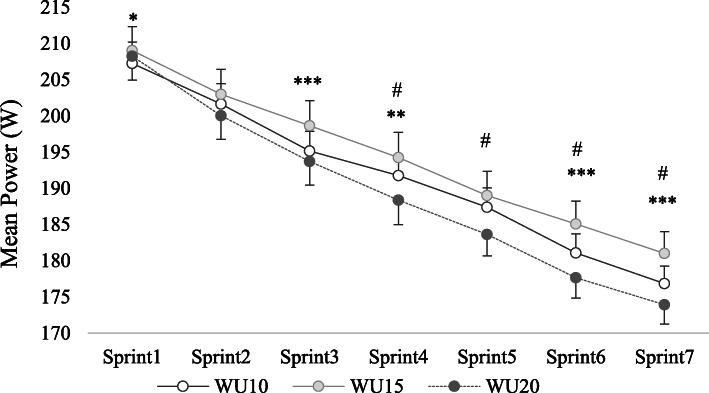
Results (mean ± SD) of the power generated in each sprint (34.2 m) recorded after WU10, WU15 and WU20 durations. * Significant difference between WU15 and WU10 at: * *p* < 0.05; ** *p* < 0.01; *** *p* < 0.001; # Significant difference between WU10 and WU20 at: # *p* < 0.001

Concerning mean power, the two-way ANOVA indicated significant main effects of WU durations and sprint number (F = 6.833; *p* = 0.005; η^2^ = 0.406; medium and F = 277.591; *p* < 0.001; η^2^ = 0.965; large, respectively). The interaction WU duration × sprint number was significant too (F = 4.767; *p* < 0.001; η^2^ = 0.323; medium). The post hoc analyzes showed: (i) compared to WU10, the WU15 leads to higher power-output recorded in all sprint repetitions, except the 2nd and the 5th one (*p* = 0.103 and *p* = 0.051, respectively), (ii) compared to WU20, the WU10 leads to higher power production during the 4th, 5th, 6th and 7th repetitions (*p* < 0.001 for all comparisons), but not for the 1st, 2nd and 3rd test repetitions (*p* = 0.229; *p* = 0.053 and *p* = 0.087 respectively; Fig. 2).

**Fig. 3:**
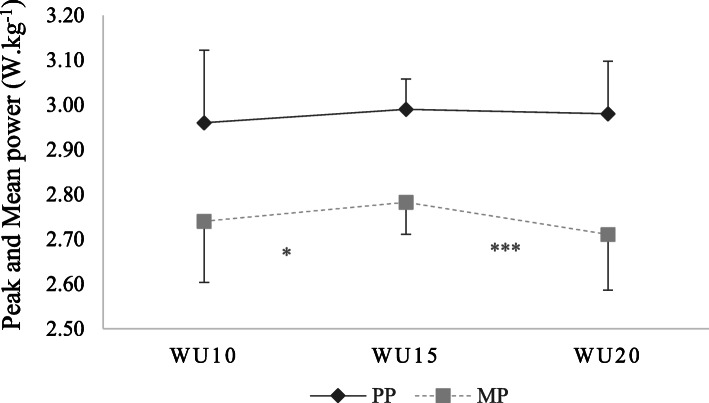
Peak (PP) and mean (MP) power per weight, recorded after WU10, WU15 and WU20 durations. * Significant difference at: * *p* < 0.05; *** *p* < 0.001

Concerning PP, the one-way ANOVA indicated that the main effect of WU durations was not significant (F = 1.028; *p* = 0.376). However, concerning MP, the one-way ANOVA indicated that the main effect of WU durations was significant (F = 6.999; *p* = 0.005; η^2^ = 0.412; medium). The Post-hoc tests showed that MP recorded after WU15 was higher in comparison to WU10 (*p* = 0.039; η^2^ = 0.240; small) and WU20 (*p* = 0.001; η^2^ = 0.674; large; Fig. 3).

Concerning FI, the one-way ANOVA indicated that the main effect of WU durations was significant (F = 11.651; *p* < 0.001; η^2^ = 0.538; large). The post-hoc test showed higher FI values after WU20 compared to WU15 (*p* < 0.001; η^2^ = 0777; large.) and WU10 (*p* = 0.004; η^2^ = 0.573; large).

### Tympanic (T_tym_) and body (MBT) temperatures

Changes from baseline ΔT_tym_ and ΔMBT at post-WU and post-RSA point of measurements, are presented in Table 1.

**Table 1:** Changes from baseline of Tympanic (ΔT_tym_) and body temperatures (ΔMBT), after the warm-up durations and the RSA

	ΔT_**tym**_ (°C)		ΔMBT (°C)	
	ΔT Post-WU	ΔT Post-RSA	ΔT Post-WU	ΔT Post-RSA
**WU10**	0.19 ± 0.34	0.81 ± 0.38^*^	0.15 ± 0.25	0.77 ± 0.28^*^
**WU15**	0.37 ± 0.21^†^	0.97 ± 0.41^#*^	0.34 ± 0.32^††#^	0.85 ± 0.35^###*^
**WU20**	0.53 ± 0.21^†††^	1.18 ± 0.38^†††*^	0.55 ± 0.20^†††^	1.11 ± 0.28^†††*^

†Significantly different from WU10 at the same point of measure at: ^†^ *p* < 0.05; ^††^ *p* < 0.01; ^†††^ *p* < 0.001

#Significantly different from WU20 at the same point of measure at: ^#^ *p* < 0.05; ^###^ *p* < 0.001

*Significantly different from ΔT post-WU at the same WU duration at: ^*^ *p* < 0.001

Concerning ΔT_tym_ and ΔMBT, the two-way ANOVA indicated that the main effects of measure were significant (F = 160.621; *p* < 0.001; η^2^ = 0.941; large, and F = 257.518; *p* < 0.001; η^2^ = 0.963; large, respectively). The main effect of duration was significant for both variables (F = 5.395; *p* = 0.013; η^2^ = 0.350; medium, and F = 8.998; *p* = 0.002; η^2^ = 0.474; medium, respectively). However, no significant interaction (duration × measure) was recorded (F = 0.113; *p* = 0.894, and F = 0.775; *p* = 0.474, respectively). The post-hoc tests revealed that ΔT_tym_ and ΔMBT at post-WU point of measure were higher after WU15 and WU20 compared to WU10 (*p* = 0.039; η^2^ = 0.173; small and *p* < 0.001; η^2^ = 0.496; medium for ΔT_tym_; *p* = 0.005; η^2^ = 0.197; small and *p* < 0.001; η^2^ = 0.692; large for ΔMBT, respectively). The ΔT_tym_ and ΔMBT at post-RSA point of measure were higher in WU20 compared to WU15 (*p* = 0.020; η^2^ = 0.187; small for ΔT_tym_; *p* < 0.001; η^2^ = 0.440; medium for ΔMBT, respectively).

### Heart rate (HR) and rating of perceived exertion (RPE)

HR and RPE scores recorded during the different experimental conditions are presented in Table 2.

**Table 2:** Heart rate and rate of perceived exertion scores at rest, after the warm up (Post-WU) and at the end of the RSA-test (Post-RSA)

		WU10	WU15	WU20
	**Rest**	68 ± 8^***^	66 ± 8^***^	67 ± 10^***^
**HR** (bpm)	**Post-WU**	130 ± 10	140 ± 10^††^	143 ± 15^†††^
	**Post- RSA**	172 ± 13	178 ± 9	179 ± 9
	**Rest**	6.3 ± 0.5^***^	6.2 ± 0.4^***^	6.4 ± 0.5^***^
**RPE**	**Post-WU**	9.4 ± 1.7	11.9 ± 0.7^†††^	12.9 ± 0.8^†††#^
	**Post- RSA**	16.3 ± 1.1	16.4 ± 0.7	17.9 ± 0.6^###^

^*^ Significant difference between rest and Post-WU values at: ^***^ *p* < 0.001

^†^ Significantly different from WU10 at the same point of measure at: ^††^*p* < 0.01; ^†††^ *p* < 0.001

^#^ Significantly different from WU15 at the same point of measure: ^#^ *p* < 0.05^; ###^ *p* < 0.001

Concerning HR, the two-way ANOVA indicated that the main effect of both measures (F = 1197.642; *p* < 0.001; η^2^ = 0.991; large) and WU durations (F = 5.248; *p* = 0.014; η^2^ = 0.344; medium) were significant, with a significant interaction (WU duration × measure; F = 4.111; *p* = 0.007; η^2^ = 0.291; small). The post-hoc tests showed that: (i) HR recorded after WU was higher than rest and lower than values recorded after the RSA test in all experimental conditions (i.e. WU10, WU15 and WU20, *p* < 0.001). (ii) HR values recorded after WU15 was higher than those recorded after WU10 (*p* = 0.021; η^2^ = 0.463; medium), while no differences were found if compared to those recorded after WU20 (*p* = 0.919).

Concerning the RPE, the two-way ANOVA indicated that the main effect of both measure and WU durations were significant (F = 1466.79; *p* < 0.001; η^2^ = 0.993; large) and (F = 31.282; *p* < 0.001; η^2^ = 0.788; large), respectively, with a significant interaction (WU duration × measure; F = 10.866; *p* < 0.001; η^2^ = 0.521; large). The post-hoc tests showed that: (i) RPE scores recorded after WU were higher than values recorded at rest but lower than those recorded after the RSA in all experimental conditions (i.e. WU10, WU15 and WU20; *p* < 0.001). (ii) RPE scores recorded after WU15 were higher than those recorded after WU10 (*p* < 0.001; η^2^ = 0.713; large), but lower than WU20 (*p* = 0.021; η^2^ = 0.478; medium). (ii) RPE scores post-RSA were higher in WU20 compared to WU15 sessions (*p* = 0.001; η^2^ = 0.641; large). However, no significant difference was recorded between WU15 and WU10 sessions in RPE scores at post-RSA point of measure (*p* = 0.828).

## Discussion

The purpose of the present investigation was to examine the effect of WU durations in a hot-dry climate (~ 30 °C and ~ 18% RH, respectively), on thermoregulation, power-output, and muscular fatigue during specific repeated-sprint ability in amateur soccer players. The main findings was that in hot climate: (i) PP was not affected by all WU durations. (ii) The WU15 in the heat leads to a higher increase in muscular power during the RSA-test. (iii) The WU10 was insufficient to induce an optimal preparedness level necessary for high-intensity effort, while the 20 WU causes higher increases in body temperature, higher RPE estimations and emergence of signs of fatigue.

The choice of WU intensity (i.e. 70% of MAV) was related to previous studies proving that warm-up at low intensities during the Raise stage (< 50% of *V*O_2max_) could be beneficial for strength and power performances [1, 32]. It was demonstrated that 10 min WU at an intensity midway between lactate inflection and lactate threshold is still appropriate for enhancing first sprint intermittent performance in hot environmental condition [33].

### Power-output (MP and PP) and core temperatures (T_tym_ and MBT)

The result of the present study showed higher MP values following WU15 duration compared to WU10 and WU20. However, no significant differences between PP values recorded after all WU durations. As the statistical power of PP was set at 1-β = 0.569, all interpretations regarding this parameter should be done carefully. Therefore, it seems that all the suggested WU durations have the tendency to induce the same effect on the best RSA sprinting speed, usually occurring in the first trial [8]. Consistent with previous researches, we found no effect of WU durations, in hot climate, on peak sprint time during the RSA. Indeed, Yaicharoen et al. [9] demonstrated no active WU effect, on first sprint intermittent performance, conducted in either thermoneutral or in hot conditions. Previously, it was reported that peak power during repeated- sprint performance was not affected by hot or humid conditions [6]. Accordingly, we suggest that the main benefits of WU on the first sprint trial during RSA seems to be more derived from temperature-related rather than non-temperature-related effects.

Moreover, the present study findings support previous researches investigating the effects of WU durations on high-intensity cycling performances reinforcing the importance of increasing the WU duration up to 15 min to reach higher morning cycling performances [16, 17, 34]. Others, suggested to reduce WU duration for intermediate and intermittent events when undertaken in hot climatic condition, to avoid an excessive increase in whole-body temperature [1]. Nonetheless, the results are still at odds with previous studies showing that there is no need to prolong WU up to 15 min before performing high-intensity exercise in the afternoon [35], or during Ramadan fasting [19]. Discrepancies between the present study findings with those of Souissi et al. [35] may be attributed to the difference in climate temperature in both investigations (30.6 ± 1.3 °C vs. 20.4 ± 1.1 °C). However, the differences in food intake and sleep habits during Ramadan period and fitness level of participants (trained amateur soccer players vs. healthy active men) may explain the aforementioned discrepancies with the study of Baklouti et al. [19].

The higher mean power-output values recorded after the WU15 in comparison with WU10 is certainly related to the longer Raise duration [1] within WU15, and may be related to the higher increase in core temperature [36, 37]. This increase in temperature was shown to be the major factor responsible for improving the nerve conduction velocity, the enzymatic activities [38], the oxygen delivery to muscles and the decreased muscular viscous resistance [39]. Likewise, it was shown that an increase in muscle temperature could be responsible for ~ 4% improvement of muscular leg power for each 1 °C elevated [32]. Moreover, it was demonstrated that at the onset of moderate-intensity exercise (WU procedures), muscular temperature increases rapidly within 3-5 min, and reaches a plateau after 10-20 min [40]. The changes from baseline of T_tym_ and MBT recorded after the WU15 and WU20 durations, seem to be similar to the values shown in the study of Yaicharoen et al. [33], recorded after active 10 min WU (intensity ~ 55% *V*O_2max_) in the heat (~ 35.8 °C), and estimated at ~ 0.3 °C. However, those changes are still inferior to the temperature variations in Drust et al. [6], recorded after an intermittent cycling effort (intensity ~ 60% *V*O_2max_) in hot environment (~ 40 °C), and estimated at ~ 1.1 °C after 10 min; ~ 1.2 °C after 15 min; and ~ 1.5 °C after 20 min of exercise. The differences in temperature values may be related to the used measuring methods (i.e. temperature pills [33], esophageal temperature [6], tympanic and non-contact method in the present study) or to the differences in environment temperatures. Notwithstanding, the accuracy and validity of non-invasive thermometers was criticized [41], recent studies recommended the use of tympanic and non-contact skin methods during and after exercise in the heat [41, 42]. The present study reveals increases in tympanic and body temperature at the end of all WU durations with more relevant increases observed after the WU15 and WU20 durations compared to WU10. Nonetheless, the higher increases of MBT following WU20 compared to WU15 indicate that the temperature cannot be the only raison to improve muscle’s power and shows no causal relationship between the two processes: temperature increase and repeated-sprint ability. While T_tym_ and MBT used in this investigation reflect more the peripheral rather than central temperature, we can assume that the increase in T_tym_ and MBT is the result of metabolic heat production from active skeletal muscle contraction. Previously, it was reported that dynamic muscle contraction produces heat, responsible for muscle temperature increases and then changes in core temperature [1, 43].

### Fatigue index (FI) and rating of perceived exertion (RPE)

Several studies reported no effect of WU duration on fatigue index (FI) computed after the 30 s Wingate-test with or without a recovery rest interval separating it to performance [16, 17, 37]. However, it was shown recently, that 15 and 20 min cycling warm-up (at 50% of MAP) induce higher RPE and FI values during 30 s Wingate-test when compared to 5 min duration in physical education students [44]. To the author’s best knowledge, this is one of the few studies showing an effect of WU duration on FI. The discrepancies with previous opposed studies may be attributed to the chosen sample size, to the training experiences or to the strategy used by participants to distribute work and energy expenditure throughout high-intensity exercise (Pacing strategy) [18, 36]. It should be acknowledged that fatigue is complex, and it is determined by the interplay between psychological (motivation) and physiological (metabolic) factors of both peripheral and central origin [3].

In the present study, the higher power decrement after WU20 compared to WU10 and WU15 is related to the drop in power starting from the 3rd and continuing to the last sprint repetitions, and was reflected by the higher RPE estimations during the WU20 session. Therefore, FI seems to be more related to metabolic and motivational changes, generating power drop during RSA, rather than to an excessive increase in core temperature. Previously, it was demonstrated that exercise in the heat leads to a greater reliance on muscle glycogen, anaerobic metabolism, muscle and blood lactate accumulation [4], generating fatigue and a decline in force production. Moreover, high-intensity exercise in the heat causes relevant impairment in oxygen delivery to the exercising muscles related to cardiac and muscle blood flow decreases [45].

Nonetheless, some limitations occur in the present study: first, for performing measurement of skin temperatures by digital thermometer and avoiding the sweat effect, the researchers used a soft cotton swab dipped in alcohol to clean the four sites: C, D, H and J chosen (see the Methods section). The skin was allowed to dry completely before checking the temperature, and this could have changed slightly the measured values. Second, the weather conditions were only controlled by measuring the ambient temperature and the relative humidity. As wind speed could have an effect on RSA and MST measurements, we canceled all sessions in which we thought that the wind speed might influence our measurements. Despite these limitations, the present study has meaningful implications for soccer players competing in hot climate temperature with relatively reduced humidity, characterizing the harsh desert climate of the Eastern Province in Saudi Arabia.

Taking into account that several competition events may hold in more challenging environments, with higher temperatures (> 40 °C), higher relative humidity (> 80%), or relative hypoxia, it is stills interesting to focus in future studies on those orientations. More topics that are interesting may also concern the combination of both heat stress and hypoxia [2]. The effect of heat/hypoxic stress on power output and fatigue after high-intensity effort seems to be an interesting perspective, allowing better understanding of fatigue complexity and the interplay between psychological (motivation) and physiological (metabolic) factors of its both peripheral and central origin [3].

## Conclusion

This study provides useful information necessary for better structuring WU when training or competing in hot-dry (~ 30 °C; 18% RH) climate conditions. The WU durations affect the mean power-output during RSA, but not peak power. The WU15 conducted at 70% of MAV and generating an RPE estimation ~ 11-12, appears an optimal pre-conditioning duration that better assists mean power-output in specific soccer repeated-sprint ability. Reducing WU duration up to 10 min does not improve power-output, while augmenting it up to 20 min induces a drop in muscular power and an increases in fatigue. Therefore, it is important that coaches and soccer players individually adapt warm-up durations prior to competitions and training sessions, to optimize physiological responses and performances in a hot climate.

## Data Availability

The data collected and analyzed in the present study are not publicly available due to ethical restrictions, but are available from the corresponding author upon request.

## Abbreviations

WU: Warm-up
RSA: Repeated-sprint ability
PP: Peak power
MP: Mean power
FI: Fatigue index
T_tym_: Tympanic temperature
MST: Mean skin temperature
MBT: Mean body temperature
RPE: Rating of perceived exertion
DS: Dynamic stretching
MAV: Maximal aerobic velocity
MAP: Maximal aerobic power
R: Rest
S: Sprint
